# Forecasting individual exercise sweat losses from forecast air temperature and energy expenditure

**DOI:** 10.3389/fspor.2023.1277070

**Published:** 2023-12-04

**Authors:** Samuel N. Cheuvront, Kurt J. Sollanek, Robert W. Kenefick

**Affiliations:** ^1^ Sports Science Synergy, LLC, Franklin, MA, United States; ^2^ Department of Kinesiology, Sonoma State University, Rohnert Park, CA, United States; ^3^ Entrinsic bioscience, LLC, Norwood, MA, United States

**Keywords:** dehydration, hyponatremia, fluid intake, exercise fluid needs, H2Q algorithm

## Abstract

**Introduction:**

Recent success in predicting individual sweat losses from air temperature and energy expenditure measurements suggests a potential for forecasting individual sweat losses for future combinations of environment and exercise. The purpose of this study is to determine the plausibility of accurately forecasting exercise sweat losses from meteorological air temperature forecasts and individual running energy expenditure forecasts. The potential impact on plasma sodium is also estimated when setting drinking rates equal to forecast sweat losses.

**Materials and methods:**

Individual exercise sweat losses (equated to water needs) and energy expended while running were measured in 33 participants along with air temperature and compared with forecasts of the same. Forecast inputs were used in a web app to forecast exercise sweat losses for comparison with observed values. The bias between forecast and observed exercise sweat losses was used to calculate the potential drinking impact on plasma sodium.

**Results:**

The concordance correlation coefficient between forecast and observed values was 0.95, 0.96, and 0.91 for air temperature, energy expenditure, and exercise sweat losses, respectively, indicating excellent agreement and no significant differences observed via *t*-test. Perfect matching of water intake to sweat losses would lower plasma sodium concentrations from 140 to 138 mmol/L; calculations using the 95% limits of agreement for bias showed that drinking according to forecast exercise sweat losses would alter plasma sodium concentrations from 140 to between 136 and 141 mmol/L.

**Conclusions:**

The outcomes support the strong potential for accurately forecasting exercise sweat losses from commonly available meteorological air temperature forecasts and energy expenditure from forecast running distance.

## Introduction

Differences in diet and activity among individuals creates uncertainty around setting a single water requirement for health and performance. Therefore, research aimed at solving the problem of individual variation in water needs is recommended (1, 2). Activity can significantly increase daily water needs in direct proportion to the exercise sweat losses (SL) that afford body heat balance through evaporative cooling (3). Fluid replacement strategies can also interact with variability in sweat salt losses and exercise duration to create concerns over plasma sodium maintenance (4, 5). An accurate prediction of individual exercise sweat losses may, therefore, assist in defining individual water needs more precisely and assuage risks around under- and over-drinking. Although sweat prediction models can be satisfactory for the purposes of group water planning in occupational (6), military (7), and sports medicine settings (8, 9), only one patented (10) algorithm has ever demonstrated sweat prediction accuracy at the individual level (11).

Sollanek et al. (11) demonstrated that knowledge of air temperature and individual energy expended when running could be used to predict *post hoc* sweat losses with accuracy on par with scale weighing. To plan for individual water needs *a priori*, such as how much water to carry on a hike through the Grand Canyon or how many water stops to make for an upcoming marathon, both air temperature and energy expenditure must be forecasted. A knowledge of air temperature seems plausible given that this information is forecasted to within ±1°C accuracy up to 72 h in advance (12–14). Furthermore, the energy cost of planned movement can be reasonably estimated for many activities (15, 16). It has been speculated (9, 11) that the same algorithmic approach taken previously could be used to theoretically forecast sweat losses and water needs at the individual level, assuming accurate forecast inputs.

The purpose of this brief research report was to determine the plausibility of accurately forecasting exercise sweat losses from meteorological air temperature forecasts and estimates of energy expenditure for track running. A secondary purpose was to assess the risk of drinking in accordance with forecasts on plasma sodium concentrations. Our hypothesis was that air temperature and energy expenditure forecasts would be sufficiently accurate to permit accurate individual exercise sweat loss forecasts that support the maintenance of plasma sodium when drinking in accordance with forecast losses.

## Materials and methods

This brief research report involves a secondary analysis and modeling of data collected and published by Sollanek et al. (11). Briefly, participants gave written informed consent that followed the guidelines of the Sonoma State University Institutional Review Board, and experiments were performed in accordance with the ethical standards of the Helsinki Declaration. Targeted down selection of the data set to runners completing ∼1 h of exercise was done so that SL (L) and sweat rate (SR, L/h) were the same. The purpose of this matching was to account for the time it takes for the sweat rate to reach a steady state, thus ensuring that the sweat rate measured in the first hour could be reasonably applied to the second hour of exercise for modeling purposes (see Forecast data, below). The 33 recreational runners, 2 of whom participated twice for a total of 35 individual observations, included 14 men and 19 women between the ages of 17 and 52. The participants ran a mean (SD) of 59.4 (0.6) min outdoors on a standard 400 m track and covered 10.2 (1.4) km.

Measured data: Testing took place in Northern California (38°N, 123°W) between the months of February and September to obtain sweat data for both cool and warm air temperatures. The environmental conditions were continuously collected and averaged hourly using a portable wet bulb globe temperature monitor (Kestrel 4400; Nielsen-Kellerman, Boothwyn, PA, USA) positioned on a rotating tripod vane placed precisely at 1.2 vertical meters from the track surface (13). Energy expenditure when running was measured using open-circuit-expired gas collection (TrueOne 2400; Parvo Medics, Sandy, UT, USA) on a motor-driven treadmill. The software within the Parvo Medics system was used for automated energy conversions using the volume of oxygen per unit of time and the non-protein energy equivalent of oxygen at a given respiratory quotient. After a 5-min warm-up consisting of a slow walk or jog, 10 min of running was completed at ∼80% of each runner's personal best 10 km race pace so that 60 min of track running at the same speed could be completed comfortably. Respiratory data were collected at 30 s intervals, and Minutes 7 through 10 were used to calculate steady-state energy expenditure. A 1% grade was used to better reflect the energy cost of over-ground running (17). As a check, a small subset of runners (*n *= 8) undertook an identical treadmill test, but at 0% grade. The higher energy cost of a 1% grade was consistent with the added cost of overcoming air resistance (18) (data not provided). Outdoor running on a flat track at treadmill pace was therefore considered convergent with energy expenditure measures indoors (17). Participant sweat losses were calculated from the seminude baseline body mass and the post-run body mass, corrected for non-sweat mass losses using lab-measured energy expenditure and allowing for recovery (11, 19, 20).

Forecast data: Air temperature forecasts could not be obtained after the fact for historical test days and times, and therefore, to simulate air temperature forecast errors, the 35 Kestrel-measured air temperatures in this study were assigned random forecast errors according to Cheuvront et al. (13). Briefly, the individual bias between 35 pairs of 72-h forecast air temperatures and Kestrel-measured air temperatures was calculated. Bias was linear across a wide range of air temperatures (13); therefore, individual differences between forecast and measured air temperatures were randomly assigned to the 35 Kestrel observations in Sollanek et al. (11) to represent potential forecast errors. Energy expenditure forecasts were made by estimating the energy cost of running (1 kcal/kg/km) from the product of body mass (kg) and planned running distance (km) (21). Measured and forecast energy expenditures were expressed as a rate, and the duration of exercise in Sollanek et al. (11) was doubled to model potential sweat and drinking accumulation errors over time. Observed sweat losses were compared with linear, steady-state predictions using the patented H2Q algorithm (10) used by Sollanek et al. (11), which is a steady-state evaporative heat balance model. Any errors produced by the delayed on and off kinetics of sweating are presumed small or to be canceled out in the practical weighing methods required to perform a validation (19). Kinetics modeling is used in combination with carefully measured sweat losses to illustrate this point (please see Appendix). Exercise sweat losses were forecast by inserting forecast air temperature and energy expenditure into a web-based algorithm (10) (https://webapp.sportssciencesynergy.com). All web-based calculations were independently performed by two investigators (SC and KS) to cross-check and ensure that no errors occurred in data entry or extraction. The web-based application was created using black box engineering (Sequoia Applied Technologies, Inc., Sunnyvale, CA, USA) to obscure the proprietary algorithm equation coefficients (10) while retaining input–output functionality. Exercise sweat losses were equated with required drink volumes 1:1 for the purposes of this study. All observed and forecast sweat losses were expressed as a rate and extrapolated to 120 min of exercise as described above for energy expenditure to model potential drinking needs accumulation errors over time.

To understand the magnitude of under- or over-forecasting of exercise sweat losses as a proxy for water needs, and as a complement to statistical outcomes, potential plasma sodium concentration or dilution effects of under- or overdrinking based on forecasts were examined (22). Briefly, the 66.7 (11.7) kg mean body mass of the study participants was multiplied by 0.60 to estimate total body water (TBW), or ∼40 L. The bias and 95% limits of agreement (LoA) in forecast sweat losses were added or subtracted from TBW to solve for plasma sodium ([Na]p2) concentration or dilution in accordance with Eq. (22):

$$\left[\mathrm{Na}\right]p2=\left(\frac{\left(\left[\mathrm{Na}\right]p1+23.8\right)\times \mathrm{TBW}i+1.03\Delta \left(\mathrm{Na}+K\right)}{\mathrm{TBW}i\;\pm \;\mathrm{bias}}\right)-23.8,$$

where [Na^+^]p1 = 140 mmol/L; TBW*i* = 40 L; Δ(Na^+^ + K^+^) are the estimated unreplaced losses of sweat electrolytes assuming common values of 40 mmol/L (Na^+^) and 4 mmol/L (K^+^), respectively, multiplied by total sweat loss (L), and ±bias is the forecast sweat loss (expressed as a drinking volume) deficit or surfeit. This approach yielded a single *average participant* for modeling.

Descriptive data are described using the mean and standard deviation (SD). Comparison data are presented using the mean and 95% LoA, calculated as the product of 1.96 and the SD of the bias, which, itself, is calculated as forecast value minus observed value, so that over- and underforecast values are positive and negative, respectively. All data conformed to parametric analyses. Quantitative agreement between observed and forecast values was assessed using the concordance correlation coefficient (CCC), which measures the degree of departure between observed and forecast values relative to perfect concordance, or line of identity, rather than the best-fit line of prediction (i.e., ordinary regression) (23), since the former is more practical and easily interpretable. Between 10 and 20 data pairs are recommended for use with the CCC (23); therefore, a plot of 35 data pairs was considered adequate for a meaningful interpretation of results, whereby a CCC > 0.80 is considered excellent agreement (24). Quantitative differences between measured and forecast 120 min accumulated sweat losses were compared by using the *t*-test. The 35 paired observations were determined satisfactory with a moderate-to-large effect size of ≥0.70, assuming conventional alpha (0.05) and beta (0.20) values. Individual error was assessed using the percentage concordance, calculated as [concordant pairs/(concordant pairs + discordant pairs)] × 100, where concordance occurs when the accumulated error is less than 500 ml (i.e., 250 ml/h) (11). Statistical analyses were performed and graphical displays were created using GraphPad Prism version 9.5 (GraphPad Software, La Jolla, CA, USA, www.graphpad.com).

## Results

**Figure 1:**
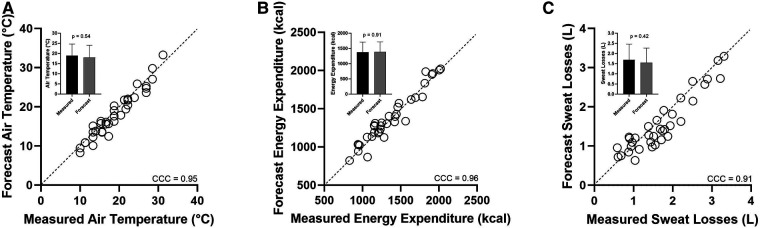
(**A**–**C**) Regression plot of forecast (*y*) vs. measured (*x*): (**A**) air temperature, (**B**) energy expenditure, and (**C**) exercise sweat losses The dashed diagonal lines represent perfect concordance (line of identity). CCC is the concordance correlation coefficient. The histogram insets indicate *t*-test results.

The observed air temperature was 19°C (6.0). The 72-h forecast errors applied according to Cheuvront et al. (13) produced a −1.0°C bias (−4, 2), which is typical of forecasts (12, 14). Absolute forecast values were 18°C (6.0). Figure 1 illustrates excellent agreement (CCC = 0.95), with no significant differences between observed and forecast air temperatures (Figure 1A inset). The forecast energy expenditure for running, estimated as 1 kcal/kg/km (21), was remarkably close to the mean measured cost of running, 0.99 (0.07) kcal/kg/km, but individually, over- or underforecast the observed value by 11%–23%, respectively. However, the agreement between observed and forecast energy expenditure remained excellent (CCC = 0.96), with only a small bias (<1%) of 9 kcal (−165, 184) (*p* > 0.05; Figure 1B inset) over 120 min of running.

Forecast sweat losses using the H2Q algorithm (10) applied *post hoc* by Sollanek et al. (11) are subject to any compounding errors related to air temperature and energy expenditure forecasts, but the agreement was still high (CCC = 0.91) (Figure 1C) (*p* > 0.05). Measured sweat losses were 1.69 (0.76) L and forecast losses were 1.55 (0.71) L. The bias was −0.143 L (−0.700, 0.414) L during 120 min of running. The percentage concordance was 88.5% (31/35 with <500 ml error). Using the equation of Kurtz and Nyguen (22), a perfect matching between sweat loss and pure water intake was calculated to reduce plasma sodium concentrations from 140 to 138 mmol/L due to the loss of sweat solute coupled with 100% water replacement. The small negative forecast bias (−0.143 L) resulted in a plasma sodium estimate of 139 mmol/L. Applying the LoA for bias to the equation, it was found that the most extreme plasma sodium concentrations ranged from 136 to 141 mmol/L, which is still within typically reported reference ranges for plasma sodium. To determine whether modeling of the *average participant* might underestimate the matched drinking impact on the smallest of endurance runners, we also modeled a 50 kg person with 50% TBW. Applying the same average sweat loss volume (1.69 L or 0.845 L/h) and 95% LoA within the Kurtz and Nyguen (22) equation as described above, the plasma sodium level ranged similarly from 134 to 142 mmol/L.

## Discussion

The purpose of this brief report was to determine the potential for accurately forecasting exercise sweat losses from meteorological air temperature forecasts and estimates of energy expenditure for planned running distance. A secondary purpose was to assess the potential forecast drinking impact on plasma sodium concentrations. The results indicate an excellent agreement between the observed exercise sweat losses and the forecasted losses when running outdoors, in a range of cool and warm environments for 120 min. Furthermore, drinking in accordance with forecast sweat losses did not increase the risk of over- or underdrinking. The outcomes support a strong potential for forecasting exercise sweat losses from commonly available meteorological and physiological data.

The ability to accurately forecast air temperature and energy expenditure (Figures 1A,B) is key to accurately forecasting sweat losses and water needs. Indeed, the influence of air temperature and energy expenditure on sweat losses and water needs is fundamental (1, 25). The explanations for this fact lie bundled within physiology and biophysics (26) and can be modeled for practical use in accurately estimating exercise sweat losses at the individual level *post hoc* (11) and now *a priori* (Figure 1C). Although the air temperature forecasting errors were applied retrospectively in this study, rather than prospectively, as would be done in practice, the average ± 1°C accuracy of forecast air temperatures (Figure 1A) is consistent with those of modern meteorology (12–14) and the common performance use of weather app technologies by consumers for the purposes of planning future activities outdoors.

The average energy expenditure error was <1%, which is well below the ∼10% commonly reported for many types of wearable technologies (27) and when compared with an indirect calorimetry gold standard such as the one used by Sollanek et al. (11). The excellent observed agreement (Figure 1B) may be explained in part by the simplicity of the exercise performed (i.e., running on a flat surface at a constant pace). However, the energy cost of movement for many activities can be accurately estimated by performing careful measurements of the body mass and distance covered (15) or characterized using MET values for similar purposes (16). This approach has also been used successfully for sports other than running, but only for groups (9). More challenging weather conditions, such as combinations of higher heat, humidity, and solar heat load, will intuitively lead to greater forecast challenges. The effects of non-steady-state running, and more extreme topography (e.g., trail running), as well as different sports, must also be considered in the future (9), but the potential for forecasting exercise sweat losses as described herein remains very convincing.

The importance of the bias measured between forecast and observed exercise sweat losses was assessed by calculating the dilution (overdrinking) or concentration (underdrinking) potential of the bias on plasma sodium concentrations, which is one important health or performance risk related to drinking strategy efficacy (2, 5). Even when considering the 95% LoA for the forecast sweat loss bias, plasma sodium concentrations were estimated to fluctuate by −4 to 1 mmol/L, where perfect forecasting would have produced a drop by −2 mmol/L. Although a smaller person was modeled to experience a −6 mmol/L drop in plasma sodium, it is important to remember that these estimates assume no solute intake and no urination over 120 min, and ordinary plasma sodium concentrations to start. In this example, they also assume that runners will drink precisely what was forecast, which may not be feasible or strictly advisable during exercise (2, 3) but, nevertheless, does inform the runner of how much water must ultimately be replaced to achieve fluid balance. The equation used (22) is well established and has been validated by an empirical measurement performed during exercise in the heat (28). Therefore, the practical efficacy of the results is sound.

A previous application of the algorithm (11) was to use air temperature and energy expenditure inputs on offer from most wearable sports devices to assist athletes with personalizing hydration recommendations *post hoc*. Exercise sweat losses are an important feedback parameter for daily training and competing as they afford insights into drinking needs and daily fluid balance. *A priori* forecast information like that presented herein may provide another benefit of water planning around training and race-day strategies around drinking. For example, the error associated with sweat loss forecasts over 2-h of running were <500 ml in 31/35 subjects (a mean bias of −143 ml). Smith et al. (29) have shown that on different days in the same environment, the level of bias in individual 2-h sweat losses can exceed 500 ml; in different environments, still temperate by most standards, sweat loss bias over the same duration can reach a level of 1,000 ml, the latter observation a well known phenomenon (25). Therefore, sweat loss forecasts may be as accurate as scale weighing, findings similar to observations made for *post hoc* predictions (11). Of even greater value is the accuracy of forecasts over a wide range of possible air temperatures and exercise intensities. The algorithm could obviate the need for careful, but impractical or impossible, body mass measurements across myriad conditions and potentially eliminate guesswork around training and race-day drink volume planning. For exercise of more extreme duration (>2 h), personalized knowledge of sweat electrolyte composition may also be of great value (4, 5).

This brief research report determined the potential for accurately forecasting individual exercise sweat losses from meteorological air temperature forecasts and estimates of energy expenditure for planned running. The results indicate an excellent agreement between observed and forecast values when running outdoors in a range of cool and warm environments for 120 min. Further, these forecasts do not increase over- or under-drinking risk, producing instead normal fluctuations in plasma sodium when drinking in accordance with forecast sweat losses. The outcomes, therefore, support the strong potential for forecasting individual exercise sweat losses from commonly available meteorological and physiological data forecasts when using the H2Q algorithm (10, 11).

## Figures and Tables

**Figure A1:**
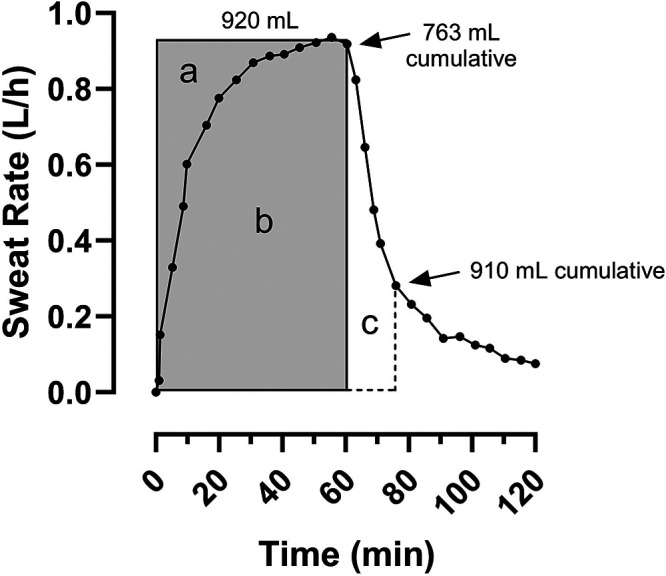
Sweating rate (L/h) on- and off-kinetics modeled after Gagnon and Kenny (32) EHL data.

## Data Availability

The raw data supporting the conclusions of this article will be made available by the authors, without undue reservation.

## Appendix

Sweating kinetics follow a similar pattern to other thermoregulatory responses to exercise (e.g., 30) and can be carefully quantified using a Potter Balance (31), direct calorimeter (32), or similar technologies. Open source software (https://plotdigitizer.com) was used to extract data from Figure 6D in Gagnon and Kenny (32), who used a direct calorimeter to measure evaporative heat loss (EHL) across time. The graph was then reverse-engineered from the numbers and fitted to a one-phase exponential association model (on kinetics) and a one-phase exponential decay model (off kinetics) to calculate precise accumulated sweat volumes across time. EHL (W) was converted to a volume sweat rate (L/h) by division using the heat of sweat evaporation (33). Evaporative efficiency of 100% was assumed (32).

Figure A1 shows sweating rate on and off kinetics compared with estimated steady-state (square wave) sweating rates required for heat balance (a + b). The steady-state estimate (∼920 ml/h) overestimates the 60-min accumulation value (∼763 ml; b) because of the onset lag, but allowing 15 min before making a postexercise mass change measurement (19) brings the accumulation value much closer (∼910 ml; b + c) to the heat balance steady-state estimate. Even 10 min of recovery results in 884 ml, a difference of only 36 ml for an hour of exercise at a sweat rate reasonably associated with running and other sports (∼1.0 L/h) (3, 34).
